# First Year’s Work Experiences of Foreign Educated Nurses Coming to Norway From Other European Countries

**DOI:** 10.1177/2377960820970003

**Published:** 2020-11-10

**Authors:** Eva Merethe Solum, Berit Viken, Anne Lyberg

**Affiliations:** Faculty of Health and Social Sciences, University College of South-Eastern Norway, Kongsberg, Norway

**Keywords:** communication, EU/EEA, foreign educated nurses, professional competence, recruitment agencies

## Abstract

**Introduction:**

Nurses educated in the European Union and European Economic Area are automatically given professional authorization to work in all member states, facilitating workforce mobility between countries. Along with many other European countries, Norway faces nursing shortages in healthcare. European Foreign Educated Nurses are often recruited to work in Norway by agencies or apply for work themselves.

**Aims:**

To explore the experiences Foreign Educated Nurses from European Union and European Economic Area had with their preparation and orientation programs and their first year of work in Norwegian elderly care institutions

**Methods:**

The study followed a qualitative explorative design. Nine open, dialogue-based, semi-structured interviews were conducted with Foreign Educated Nurses from Poland, Lithuania, Latvia, Iceland, and Spain. Data were analyzed using qualitative content analysis.

**Findings:**

One main theme, struggling to adjust to professional competence standards, and four subthemes emerged from our data (1) deficiencies in preparation and orientation by recruitment agencies and institutions, (2) language skills and communication challenges at work, (3) cultural differences in the nursing role in clinical practice, and (4) social interactions at work.

**Conclusion:**

More comprehensive preparation and orientation programs regarding language skills and local healthcare systems are needed. Foreign Educated Nurses make important contributions to the Norwegian healthcare work force, but the challenges brought to light in this study negatively affected their work conditions and can possibly threaten patient safety. More research is suggested to address the lack of collaboration between agencies, healthcare institutions, and other stakeholders in establishing professional standards and appropriate support for Foreign Educated Nurses from European Union and European Economic Area.

## Background

The globalization of healthcare has led to an increasingly interconnected worldwide nursing workforce (Jones & Sherwood, 2014). In recent decades, there has been a tremendous change in the trends and effects of nurse mobility. The migration and internationalization of the nursing workforce has been considered a global concern which brings both challenges and possibilities (An et al., 2016; Munkejord & Tingvold, 2019; Viken et al., 2018). National and community-based expectations for countries to put both recruitment and employment procedures in place to ensure safe, competent, and ethical care are stated in the World Health Organization’s (WHO) Global Code of Practice on the International Recruitment of Health Personnel (WHO, 2010). If recruitment is well-managed, the international migration of nurses can make a comprehensive contribution to developing and strengthening healthcare systems. For this to happen, it is imperative that nurses are offered sufficient orientation programs, enabling them to conduct safe and effective nursing practices in the country where they are offered work (Munkejord & Tingvold, 2019; Viken et al., 2018). In their Position Statement on the Scope of Nursing Practice, the International Council of Nurses (ICN) holds that all employers have the responsibility to support nurses in performing their duties within the full scope of their practice. Furthermore, nurses cannot be asked to perform duties beyond the scope of their practice or competencies (ICN, 2013).

Many European Union (EU) and European Economic Area (EEA) member states are struggling with critical healthcare workforce shortages, primarily related to demographic changes, such as growing and aging populations with multi-morbidity and polypharmacy (Buchan et al., 2014; Hjemås et al., 2019; Li et al., 2014; Munkejord & Tingvold, 2019). To face such shortages, many countries in Europe, including Norway, rely on Foreign Educated Nurses (FENs) from countries within and outside the EU/EEA (Dahl et al., 2017; Scammell, 2016). Free worker movement within the EU/EEA member states facilitates the globalization of the nursing workforce. According to the Professional Qualifications Directive of the European Parliament, there is a system for automatic recognition of the professional qualifications of nurses from these countries (European Union, 2005; Glinos et al., 2014; Norwegian Directorate of Health, 2018). Furthermore, this increased mobility is also due to the active recruitment of nurses within these countries to fill vacancies (Knutsen et al., 2020; Leone et al., 2016).

Norway is among the Northern European countries with the highest proportion of FENs. The need for recruitment of FENs in Norway is partly due to demographic changes and a growing demand for healthcare in the population (Norwegian Directorate of Health, 2018). The Norwegian Directorate of Health (2018) reported that in 2016, FENs comprised 6% of the nursing workforce and one out of ten nursing authorizations was given to nurses from EU/EEA countries, excluding Nordic countries.

At the individual level, newly educated Norwegian nurses may consider working in elderly care to be a low-status position, which may be one reason for staff shortages (Johannessen, 2004). Contrastingly, nurses from Eastern European countries may regard elderly care in Norway as an attractive job position, with higher wages, more possibilities for professional development, and better regulated work hours than in their home countries (Isaksen, 2012; Knutsen et al., 2020).

Many FENs from EU/EEA countries are recruited to work for private agencies to fill vacancies in Norwegian healthcare. These agencies have contracts with nearly all Norwegian municipalities and offer FENs temporary employment in healthcare institutions (By, 2018). The Norwegian Regulations for Authorization of Health Personnel state that for nurses educated within the EU/EEA, it is the responsibility of the employer and the nurses themselves to confirm that nurses’ language skills are sufficient to provide responsible communication and care (Ministry of Health and Care Services, 2008; Norwegian Directorate of Health, 2019). That is, it is necessary to implement non-technical patient safety skills both at the system and individual levels. Non-technical skills include communication, teamwork cooperation, situational awareness, and decision-making, which are crucial for preventing harm and maintaining patient safety (Flin et al., 2013; White, 2012). In contrast with FENs from the EU/EEA, nurses educated outside the EU/EEA undergo a comprehensive process that entails working as nurse assistants and passing Norwegian language, health system, and medication tests before they can get accreditation as nurses by the Norwegian authorities (Norwegian Directorate of Health, 2019).

International studies on FENs’ experiences have revealed problems related to language, culture, and perception of nursing care tasks (Viken et al., 2018; Moyce et al., 2016). Adeniran et al. (2008) argued that FENs’ challenges during their transition to the US seem to be more related to language, communication, and socio-cultural differences than to a lack of clinical knowledge and skills. Habermann and Stagge (2010) explored the impact of FENs on professional standards of nursing care; along with other studies, they concluded that FENs recruitment needs to consider quality and safety issues in healthcare settings (Moyce et al., 2016; Sherwood & Shaffer, 2014). Moreover, Viken et al. (2018) found that longer orientation periods with continual clinical supervision are necessary, including systematic reflection on practice experiences, to support nurses in the transition period and strengthen their Patient Safety Competencies.

Few studies have been conducted on the early work experiences of those coming from within the EU/EEA to fill nursing positions. Studies from the Netherlands and Germany, where the governments encourage recruitment from other European countries, show that measures taken so far to ensure sustainable recruitment strategies need to be improved (de Veer et al., 2004; Kuhlmann & Jensen, 2015). This indicates a knowledge gap on the early work experiences of FENs. Thus, more studies are needed to better understand the challenges met by FENs in the transition period to nursing practice within the EU/EEA. Most FENs in Norway have been recruited to work in long-term care facilities for the elderly or in home-based care, where the number of vacancies is especially high (Gautun & Syse, 2017; Hjemås et al., 2019).

Therefore, this study aims to explore the experiences FENs from EU/EEA had with their preparation and orientation programs and their first year of work in Norwegian elderly care institutions.

## Methods

### Study Design

The present study followed a qualitative explorative design to achieve a better understanding of FENs’ experiences with their preparation courses and orientation programs and during their first year of work in Norwegian elderly care institutions. This design was selected because it would allow us to study nurses’ experiences from their own perspective and in detail (Polit & Beck, 2017).

### Participants

Purposive sampling was used to recruit the study participants. The inclusion criteria consisted of being FENs from the EU/EEA who had come to Norway to work in elderly care. Exclusion criteria were nurses educated outside Europe or other Nordic countries with similar language and culture as Norway. Managers and head nurses at four different elderly care institutions received verbal and written information regarding the study, which they passed on to FENs who met the inclusion criteria. FENs who reported interest in participating were contacted by telephone to arrange a date and time for the interview. Eleven FENs agreed to participate in the study, however two of them were excluded because of their origin outside EU/EEA. Seven of the participants included in the study had contacted Norwegian agencies in their home country or online, and two had used the opportunity of free movement to apply for a license and temporary work by themselves. They were all females and came from Poland, Lithuania, Latvia, Iceland, and Spain, and their ages ranged from 24–54 (mean age = 32) when they arrived. Their working experience in Norway ranged from two months to ten years.

### Research Ethics

The present study adhered to the Ethical Guidelines for Nursing Research in the Nordic Countries (Nordic Nurses Federation, 2003). The study protocol was registered with and approved by the Norwegian Data Protecting Services (NSD; No. 45462).

All participants were given detailed written information regarding the study’s background and purpose, and they all provided written informed consent. They were assured that their names and identities would not be disclosed, were given the authors’ contact information, and were told they had the right to withdraw from the study at any time. All data were stored in a locked and fireproof filing cabinet, and will be destroyed at the end of the study.

### Data Collection and Setting

Data were collected through open dialogue, based on a semi-structured interview guide held in a secluded quiet area at the participants’ workplace (Polit & Beck, 2017). Seven of the interviews were performed by the first author (EMS) and two by the second author (BV), independently. This enabled the authors to discuss the data collection process and gain mutual understanding.

The interviews were carried out in Norwegian, as it was the language the FENs used at work. They were tape-recorded and lasted 50–60 minutes. The interviews started with a short, informal conversation to ensure the participants had understood what they had consented to, and notes on demographic data were taken.

The semi-structured interview guide was developed by the authors. The initial question to all the participants was: “Will you please recall and reflect on your experiences during your first year of work in elderly care institutions?” The seven FENs recruited by agencies were asked to recall and reflect on their experiences with the arranged preparations programs.

Cultural sensitivity was adhered to by the interviewers having an open approach to the participants’ different cultural backgrounds. Since focus was on the participants’ nursing practice, special attention was given to cultural differences in education and their nursing role. These differences were respected and not discussed, as the participants were given room to talk freely without interruption.

Some participants’ reflections were on recent experiences, while others’ were on more distant memories, as some of them had been in Norway for many years.

The dialogue between participants and the interviewers gave the interviewers the opportunity of probing to clarify and deepen the information provided (Polit & Beck, 2017). The tape-recorded interviews were transcribed verbatim by the two interviewers.

### Analysis

The data were analyzed using qualitative content analysis, as described by Graneheim and Lundman (2004). An inductive approach driven by the text was applied, which consisted of the authors’ searching for patterns and distillations of the participants’ expressed meanings. All three authors were involved in the analysis process. Each interview was read thoroughly to obtain an impression of all the transcribed information. The first part of the analysis was carried out individually, after which the authors met on several occasions to discuss the meaning of the content and extent of saturation. This enabled them to discuss their interpretations and gain mutual understanding. The authors’ preunderstanding was based on years of international and global capacity building in nursing education and of research on migration and cultural issues.

Thereafter, the text was re-read, and significant words and key phrases concerning the FENs’ experiences were extracted, thus breaking the text down to meaning units. Meaning units, sentences, and aspects related to each other, were sorted into condensed meaning units. This involves the manifest content, that is, the visible and obvious components relevant to the aims of the study. The next step comprised condensing the meaning units while preserving their essence. These were compared and combined, and a thematic analysis of the content was undertaken. In the present study, these steps concerned interpretation on different levels of abstraction which resulted in four subthemes and one main theme. The main theme represents the final level of abstraction. An example of the qualitative content analysis is presented in Table 1.

**Table 1.** Examples of Meaning Units, Condensed Meaning Units, Subthemes and Main Theme From Content Analysis of the Data.

Main theme: Struggling to adjust to professional competence standards			
Meaning unit	Condensed meaning unit (Description close to the text)	Condensed meaning unit (Interpretation of the underlying meaning)	Subthemes
*I went to the agency office the next morning after arriving in Norway. There I was told to take an afternoon shift in home-based care the same day. I was much stressed, but was told by my agency that there was no need to wait—“the best way to learn Norwegian is in practice.” It was very scary*	Told to take an afternoon shift the day after arrival. The recruitment agency did not secure and ensure her competence. They expected the nurse to be operative in nursing practice care the day after she arrived and left the responsibility to the institution	Lack of sufficient orientation and preparation after arriving to Norway, led to uncertainty and stress that might affect nursing care negatively	Deficiencies in preparation and orientation by recruitment agencies and institutions
*The largest problem for me starting as a nurse in practice was language knowledge, or comprehension maybe. In the beginning, patients could ask me about something I thought I understood, but I didn’t. It was stressful. I should have asked more, but was afraid to do it too much. We do not always want to show our lack of understanding*	Difficulties to understand and comprehend Norwegian. Realized that she did not understand what the patient said, though she thought she did. Knew she should have asked more, but did not want to show her lack of understanding	Lack of language and comprehension skills led to communication problems with the patient. It was stressful for the nurse. The fact that she did not ask up again could have threatened patient safety	Language skills and communication challenges at work
*I worked at the intensive ward at home at an ordinary medical hospital; therefore, it was not difficult for me to do daily care tasks for the patients in Norwegian nursing homes. We were trained to assess patients’ conditions and monitor them, operate technical equipment, and perform technical procedures. However, we always had a doctor behind us*	Was confident to do daily care nursing tasks and technical procedures. Was trained to assess, monitor the patient, and use technical equipment, but they always had a doctor to consult	Felt confident in nursing care and knowledge, but was not used to take independent decisions which are necessary in Norwegian elderly care institutions as doctors are not available for consultation at all hours	Cultural differences in the nursing role in clinical practice
*I have met many Norwegians at work, but I do not have any friends among them. I am mostly with people from my own country, Poland, the Philippines, and Africa. Norwegians can be hard to reach; it is like they have fences around them*	Meet Norwegians at work, but it is hard to make friends with them. Do socialize with people from own country or other countries at work	It is difficult to become friends with the Norwegian nurses. Socializing was most common with nurses from own or other countries, which might make it more difficult to learn Norwegian language and culture	Social interactions at work

## Findings

Analysis of the interviews on EU/EEA FENs’ early experiences transitioning to a new work environment in Norway revealed one main theme and four subthemes, as illustrated in Table 1.

### Main Theme: Struggling to Adjust to Professional Competence Standards

The overall findings showed that participants struggled to adjust to the expected levels of professional competence in their first year of work. Evidence of their struggles is provided in the four subthemes and illustrated with quotes extracted from coded meaning units in the data.

### Subtheme 1: Deficiencies in Preparation and Orientation by Recruitment Agencies and Institutions

The FENs experienced various challenges in transitioning to their Norwegian workplaces during the period they were contracted with recruitment agencies. They did not feel sufficiently prepared for the responsibilities the agency expected them to perform in clinical practice after coming to Norway. Stress might have made it more difficult to learn and achieve new competencies. In the words of a participant:

I went to the agency office the next morning after arriving in Norway. There I was told to take an afternoon shift in home-based care the same day. I was much stressed, but was told by my agency that there was no need to wait—“the best way to learn Norwegian is in practice.” It was very scary.

There is significant variation in the levels of healthcare for the elderly within Europe. All nine participants stated that they lacked knowledge regarding how the Norwegian healthcare system was organized. In Norway, many multi-morbid elderly patients are treated in nursing homes, without referrals to hospitals. Most of the participants were not used to this model of care. They also stated that they had poor knowledge of Norwegian laws and regulations, such as patients’ rights when it comes to necessary coercion versus the principle of autonomy. Many of the participants reported that they were more used to making decisions on behalf of the patient.

During their contract period with the agency, which could last a year, the recruited nurses risked being sent to various places in Norway or to elderly care institutions with an acute need for nurses in temporary positions in the town or municipality where they were placed. Participants reported that it was more common to be sent to different institutions in big towns or urban municipalities than in rural areas, where they could work for months at one institution. One participant reported:

The first three weeks, I worked in 10 different places, seven different home-care bases in town, and three nursing homes. Always new patients and colleagues.

All the participants also reported large variations regarding orientation programs for nurses in temporary positions organized by the institutions. Frequent changes in the workplace could lead to inadequate information about the patients and an inability to answer other health personnel or next of kin when asked for patient information. Participants who started out in rural areas in small municipalities reported better work conditions as they felt well taken care of and helped by the auxiliary nurses at their new workplaces. Meanwhile, many felt they were given too much responsibility too quickly. Auxiliary nurses often have long experience, but they cannot professionally replace the responsibility of a nurse. One of the nurses interviewed expressed:

I got a two-week orientation program at the nursing home I started at when I came, that was it. After that, I was responsible for one ward for the rest of the summer.

Many participants reported challenges caused by lack of knowledge regarding electronic nursing records and reporting systems. Most of them reported that these systems were more comprehensive than in their home countries, and some were not familiar with them at all. Participants expressed frustration regarding not being trained or allowed to report directly in the electronic nursing records while they were working in temporary positions because they often had more responsibilities during their shift than they expected to have. One of the nurses described her experience as follows:

I did not get any training on the electronic nursing record system. They showed me where to write a daily report, but nothing more. Therefore, once when I had responsibility for a shift, and a patient had to be referred to the hospital, I didn’t know I had to write a hospital admission report. The hospital did not know where the patient came from or why he was referred. Of course, this can lead to lots of mistakes.

Some of the nurses felt they had to accept double shifts, therefore working more hours during a week than what is regulated by Norwegian laws. They reported to be exhausted by double shifts of up to 50 hours a week. This added stress to their first work period. This over-achievement may not have been initiated by the agency, but rather by the institution facing nurse shortages.

### Subtheme 2: Language Skills and Communication Challenges at Work

The FENs recruited by agencies reported significant variations in language preparation courses in their home countries, which ranged from two to six months in length. They had all passed a required language test initiated by the agency in their home country before they were assessed as competent to work as nurses in Norway. The two nurses who applied for work independently had taken Norwegian courses in their home countries and been assessed competent to work by their employer without being asked to undergo more courses or tests.

However, participants reported variations in the content and quality of the courses and assessment of their language skills. In participants’ home countries, local teachers with varied knowledge of language comprehension and cultural context often conducted the Norwegian language courses. All the participants stated that they experienced considerable communication challenges with other health personnel, patients, and patients’ relatives during their first year of clinical practice. A nurse reported:

When starting work in a nursing home, I suddenly realized that the three-month language course I had with the recruiting agency was not enough. It was a complete shock to realize my incomplete language skills.

FENs sometimes experienced a double challenge when they were unable to communicate with their mentor and required patients’ help to communicate:

I was told by the agency that I would get an orientation from a nurse at the site … A nurse from Sri Lanka received me. I could not understand her Norwegian, nor did she understand mine. Finally, a patient tried to help us with our communication … . The next day, I was given full responsibility for the afternoon shift. I never got more orientation.

Furthermore, participants reported that during the preparation courses and orientation programs they missed training on health-related language such as medical terminology and drug names, which is essential for safe nursing practice. One of the participants said:

When we had shift reports about different patients and someone addressed me with questions about them, I did not understand who and what they were talking about or what I was asked about.

All participants reported that they tried to write down everything that was said in reports and always used a dictionary. However, they felt stressed when they constantly had to ask colleagues to repeat what they said. They did not feel confident to disclose incomplete language comprehension skills, as they were afraid it could irritate other healthcare workers. For example:

The largest problem for me starting as a nurse in practice was language knowledge, or comprehension maybe. In the beginning, patients could ask me about something I thought I understood, but I didn’t. It was stressful. I should have asked more, but was afraid to do it too much. We do not always want to show our lack of understanding.

The following quote from one of the participants shows how the lack of adequate language skills led to inferior work conditions and made holistic nursing care impossible:

On my first shift at a nursing home, I was responsible for 32 patients, together with two auxiliary nurses. The only report I got, was information about times for medications, and that a man was in need of opiates at pre-set times. He died during my shift, and I did not have the language skills to express my condolences to his next of kin, or say anything comforting in the situation. I felt totally embarrassed, insensitive, and incompetent as a nurse.

All participants stated that they experienced challenges with communication on the telephone. One typical example was providing information to nurses or doctors for on-call services, as these professionals did not have any previous knowledge of a patient. It was difficult for the FENs to discuss and explain relevant clinical information when they could not see the other person or their body language. This could be particularly stressful and cause life threatening misunderstandings in an emergency situation, when a patient needs to be assessed for a possible hospital referral and decisions need to be made quickly. During afternoon and night shifts, it was often just one responsible nurse at the ward along with auxiliary nurses.

All participants reported challenges understanding Norwegian dialects: there are four main Norwegian dialects, which differ significantly regarding accent, grammar, syntax, and vocabulary. Their language preparation courses in their home countries did not prepare them for all the dialects they encountered in Norway. When the nurses started work or were sent by their agencies to Northern, Western, or Southern parts of Norway, it was hard for them to understand the local dialect. Furthermore, in elderly care, patients often have dementia or hearing problems. It can cause communication problems when a nurse with a strong accent and little experience in colloquial language has to communicate with a patient with an unfamiliar dialect. Some participants also experienced challenges in their daily work with colleagues of different nationalities, and felt it was even harder to understand and communicate with nurses from countries other than Norway.

### Subtheme 3: Cultural Differences in the Nursing Role in Clinical Practice

Participants experienced pronounced differences in the professional nursing role in Norway compared to that of their home countries, especially nurses from Eastern and Southern Europe. They reported having received little education in their home countries on elderly care and the challenges of geriatric multi-morbidity and dementia. For instance, a nurse explained:

When I studied and worked in my home country, I had no practice with nursing homes or elderly care, before I came here. We do not have it the same way as you.

However, most participants reported that they had adequate knowledge of theory and practical nursing procedures from education and practice in their home countries, but they were not used to making independent nursing decisions required for Norwegian elderly care nursing.

I worked at the intensive ward at home at an ordinary medical hospital; therefore, it was not difficult for me to do daily care tasks for the patients in Norwegian nursing homes. We were trained to assess patients’ conditions and monitor them, operate technical equipment, and perform technical procedures. However, we always had a doctor behind us.

Most of the nurses were used to a more hierarchical system of cooperation between nurses and doctors. Several participants referred to doctors as the clearly defined “boss” and their statements as “laws.” In Norway, they experienced a more egalitarian system for cooperation between doctors and nurses. In general, these differences were regarded as positive, and increased their self-esteem. For instance, one of the nurses expressed:

In my country the doctor is like “God.” We nurses are just very small and there is no cooperation; the doctor just delegates and commands. Here, we do have a very good relationship and cooperation; it makes me feel respected and worthy.

However, they also had more responsibilities than they had in their home countries. This could be stressful when they were alone on the ward during the evening, having to take action themselves with a patient whose condition had deteriorated and needed to be assessed for a hospital referral:

Our role here is much more independent in elderly care facilities than in a hospital in my country. For example, I have to assess and decide when to call the doctor or the emergency unit, and it was a shock to me when I had to assess when to give pre-ordinated opioids to a patient in my first practice in a home-care unit, without consulting a doctor.

The nursing responsibility to give information to patients and their next of kin was regarded as part of a higher level of independence, usually reserved for doctors in the participants’ home countries.

Some participants reported that they considered Norwegian nurses to be a bit too wary of conflict. They were perceived to be very nice, warm, and welcoming, but the FENs sometimes would have liked more openness and directness to avoid misconduct and clinical mistakes:

What I wish is that my Norwegian colleagues would speak up more if you do anything wrong or could do things better. We are used to colleagues being much more direct with each other in my country.

### Subtheme 4: Social Interactions at Work

The nurses also experienced challenges in social interactions at work, even if they generally reported feeling very welcomed by their Norwegian colleagues. However, social contact with Norwegian nurses varied. Some FENs experienced problems making friends with Norwegians outside work hours, as one of the participants recalled:

I have met many Norwegians at work, but I do not have any friends among them. I am mostly with people from my own country, Poland, the Philippines, and Africa. Norwegians can be hard to reach; it is like they have fences around them.

Elderly care institutions often have a multicultural staff, and one participant reported up to 15 different nationalities at the nursing home where she worked. This can be a challenge for socializing in the workplace, as some participants reported that personnel from one country tended to stick together during lunch and other breaks.

## Discussion

This study aimed to explore the experiences FENs from EU/EEA had with their preparation courses and orientation programs and their first year of work in Norwegian elderly care institutions. The findings indicate that FENs were *struggling to adjust to professional competence standards.* The implications of this study are discussed in light of other studies, with a focus on work conditions and patient safety, both on individual and system level.

It is estimated that 70–80% of mistakes in healthcare can be attributed to a breakdown in non-technical patient safety skill categories (Flin et al., 2013). Lack of adequate language, communication skills, and cultural competence can challenge FENs’ work conditions. This, in turn, can threaten patients’ safety, treatment, and well-being both ethically and practically (Dahl et al., 2017; Moyce et al., 2016; Viken et al., 2018).

Habermann and Stagge (2010) explored the impact of FENs on nursing care and professional standards. They concluded that the process of recruiting FENs does not sufficiently account for quality and safety issues in healthcare settings, and that prioritizing economic gains over training migrating nurses could have a severe impact on patient safety. These safety concerns have been highlighted in studies on how cultural and language differences can cause misunderstandings that seriously impact healthcare (Crawford et al., 2017; Moyce et al., 2016).

### Deficiencies in Preparation and Orientation by Recruitment Agencies and Institutions

Our findings indicated that the process of recruiting FENs—starting from their home countries until they were established in clinical practice in Norway—could vary in length by months, depending on recruitment agencies’ routines. The patient safety program in Norway addresses nurse employers who have a vital responsibility to improve the quality of healthcare through patient safety interventions (White Paper St. 10 (2012–2013), 2013).

Responsibility for the challenges reported should be shared between the municipalities having established contracts with recruitment agencies, recruitment agencies having employee contracts with FENs, and Norwegian healthcare institutions where FENs work (Ministry of Health and Care Services, 2008; Norwegian Directorate of Health, 2019). However, it is reasonable to believe that these three parties may not collaborate well enough to avoid challenges while nurses are contracted with an agency. As a consequence, it is unclear who takes responsibility for FENs’ adequate work conditions, orientation programs, and assessment of qualifications to ensure quality nursing care.

FENs play an important role in Norwegian elderly care institutions. In line with previous research, our study showed that FENs possessed adequate clinical competence; however, they faced challenges on communication, teamwork cooperation, situational awareness, and decision-making while adjusting to the nursing role (Munkejord & Tingvold, 2019; Viken et al., 2018). This is obvious during their first year of practice, which is clearly related to deficiencies in training, both by recruitment agencies and Norwegian institutions.

Acute shortage of nurses in elderly care institutions often resulted in interrupted orientation programs, and FENs often received responsibility for a ward or a shift too quickly. Participants reported that these working conditions led to stress and exhaustion, which can be associated with adverse nursing care performance (Flin et al., 2013; Prapanjaroensin et al., 2017). Changing workplaces multiple times a week or month often results in lack of adequate knowledge regarding the workplace, patients, and patients’ relatives. This is not in accordance with safe, holistic nursing stated in the Norwegian Code of Conduct for Nurses (2011), which is based on the International Code of Conduct for Nurses. Additionally, continuous movement of FENs obstructs reporting practices, rendering recruitment agencies unable to identify or help with nurses’ challenges. The institutions and wards where nurses fill a vacancy, will have little time to assess the FENs’ competencies, arrange adjusted orientation programs or supervision, or even provide adequate information about patients. Thus, it is virtually impossible for agencies and institutions to uphold their responsibility to support nurses in performing the full scope of their practice, according to the ICN(2013).

When findings from this study and two other studies in Europe (de Veer et al., 2004; Kuhlmann & Jensen, 2015) are compared with research on FENs migrating from outside the EU/EEA, similar challenges become apparent (Moyce et al., 2016; Munkejord & Tingvold, 2019; Viken et al., 2018). In Norway, FENs from outside the EU/EEA have to undergo a comprehensive preparation program regarding professional content and language courses before they receive work authorization from the Norwegian authorities. In contrast, FENs from the EU/EEA can benefit from the free movement policy stating that it is up to the employer to assess whether nurses’ language and professional competencies are adequate for providing safe nursing care (Munkejord, 2017; Norwegian Directorate of Health, 2018). This gap in preparation and orientation programs between EU/EEA and non-European nurses seems to be far too wide.

### Language Skills and Communication Challenges at Work

In line with other studies, the EU/EEA FENs reported that they experienced language, communication, and comprehension difficulties, even after being assessed as qualified by the recruiting agency or the institution that employed them. Nursing is based on teamwork and faulty communication can lead to dangerous misunderstandings, such as misinterpreting critical clinical data or decisions (Flin et al., 2013; Moyce et al., 2016). The ability for nurses to speak clearly and accurately with patients and colleagues requires linguistic competence, since decision-making is often based on discourse with colleagues, patients, or next of kin (Crawford et al., 2017; Tregunno et al., 2009).

Even though all the participants in our study sought clarification and validation to avoid misinterpretations due to lack of comprehension and cultural skills, they also reported that they did not always want to disclose their uncertainty. To counteract this, trusting working conditions, awareness, and willingness from Norwegian nurses to engage with FENs are required (Moyce et al., 2016; Munkejord & Tingvold, 2019). Confirming previous research, our findings provided several examples of how language problems are exacerbated when communication occurs by telephone, increasing the danger of misinterpretation because the other person’s body language cannot be seen (Raatiniemi & Mehus, 2012; Takeno, 2010).

### Cultural Differences in the Nursing Role in Clinical Practice

It is important to gain knowledge regarding local culture and nursing roles before starting nursing practice in another country (Moyce et al., 2016). A number of the participants reported that they were used to authoritarian traditions in healthcare, where doctors had strong positions in decision-making in clinical practice. The nursing role in Norway is more congruent with the independent role as described in the Norwegian Code of Conduct for Nurses (2011). This is also referred to as a “common difference” in other studies (Dahl et al., 2017; Kuhlmann & Jensen, 2015; Moyce et al., 2016). FENs’ identification with a role as doctors’ assistants might lead to uncertain expectations in clinical assessment and decision-making.

Being a nurse from another European country does not automatically ensure required knowledge of the Norwegian health and welfare system or patients’ rights. If a nurse is unfamiliar with patients’ and next of kin’s rights to co-determination and information under Norwegian laws, there is a danger that those rights could be violated (Patient and User Rights Act, 2001). This is even more important for elderly patients with varying degrees of dementia. All nurses need this knowledge to exercise responsibility for not harming the patient and to report adverse events during their shifts (Norwegian Patient Safety Program, 2011). Many of the participants had not been introduced to the electronic nursing record and report system, since they worked in temporary positions. Such access is crucial for all nurses to ensure continuity and qualitative good care. Even if participants felt well-received by Norwegian nurses, some wished for the Norwegian nurses to guide them more actively, in order to avoid uncertainty, misconduct, and mistakes in clinical practice.

### Social Relationships at Work

Good social relationships in the workplace are crucial for establishing an assertive and safe work environment (Flin et al., 2013). Many elderly care institutions have a multicultural staff in temporary or long-term positions, leading to diversity in cultures and languages. Newly arrived FENs use their energy to navigate communication challenges, in addition to acquiring knowledge about routines and patients. Participants reported this to drain their energy, which may affect patient safety and hinder nurses’ ability to establish good social relationships with colleagues.

## Strengths and Limitations of the Study

The findings of the present research should be interpreted taking into account the following strengths and limitations. Some participants commented on recent experiences, while others did so on older experiences. This could be both a strength and a limitation of the present study. For the newcomers, it was difficult to express themselves in Norwegian, and they had relatively little opportunity to evaluate their struggles or make adjustments to meet required nursing competencies. Participants who had been in Norway longer could reflect more fully on early work experiences and what consequences they had for their ability to conduct safe nursing practice. However, details from this period may have been forgotten.

The study’s trustworthiness was ensured by the concepts of credibility, dependability, confirmability, and transferability (Graneheim et al., 2017). Though it would have been preferable to include more participants in the study, the credibility was ensured by shedding light over the research question from different aspects by selecting participants from various countries, experiences, and workplaces. The data met a degree of saturation necessary for extracting sustainable meaning units, subthemes, and a main theme.

Dependability was ensured as new insight of the phenomena was achieved during the data gathering process. The interviewers struggled to assure confirmability by minimizing their preunderstanding with follow up questions and probing during the interview. The authors discussed, expanded, and adjusted their individual understanding of the transcribed data several times in the present study. As for transferability, more longitudinal individual and system level studies from various contexts are recommended to assess the impact of EU/EEA FENs’ preparedness and work conditions in their early work period, as well as their long-term consequences for healthcare outcomes. However, based on other studies (de Veer et al., 2004; Kuhlmann & Jensen, 2015), it is reasonable to believe that our findings are relevant for challenges met in other EU/EAA countries recruiting FENs from Europe.

## Conclusions and Implications for Practice

This study indicates that even though FENs from the EU/EEA felt they were well received and respected in Norway, they struggled to adjust to required professional competency standards and the nursing role in clinical practice. The recruitment agencies’ preparation programs in the nurses’ home countries mostly focused on teaching the Norwegian language, which the FENs found insufficient when they started clinical practice. The nurses felt culturally unprepared and had little knowledge regarding Norway’s healthcare system, laws, and regulations for healthcare professionals. The exhaustion and fatigue caused by language challenges and cultural differences in nursing roles could lead to social isolation. Even though FENs make important contributions to the Norwegian healthcare system, the challenges brought to light in this study negatively affected their work conditions, and could possibly threaten patient safety. Deficiencies in EU/EEA FENs’ preparation, orientation, and work conditions need to be addressed. Based on FENs’ countries of origin, different nurses require different kinds of support to strengthen their professional competencies, indicating a need for individualized preparation and orientation programs. More research is suggested to address the lack of collaboration between agencies, health institutions, and other stakeholders in establishing professional standards and appropriate support for EU/EEA FENs working in Norway.
